# Editorial: Supramolecular cancer therapeutic biomaterials

**DOI:** 10.3389/fchem.2023.1162103

**Published:** 2023-03-03

**Authors:** Yong Yao, Zhengtao Li, Ruibo Zhao

**Affiliations:** ^1^ School of Chemistry and Chemical Engineering, Nantong University, Nantong, China; ^2^ Department of Chemistry, National University of Singapore, Singapore, Singapore; ^3^ Department of Materials, Imperial College London, London, United Kingdom

**Keywords:** supramolecular chemistry, host-guest interaction, macrocyclic compounds, biomaterials, cancer therapy

Cancer poses a serious threat to human health (Helmink et al., 2019). In the field of cancer treatment, the commonly used methods include surgery, chemotherapy, and radiotherapy. However, these methods have application limitations, which can prevent the tumor treatment from having the desired results. (Haumann et al., 2020; Dai et al., 2021). Under this context, supramolecular therapeutic materials have been developed and have shown high application value in the early diagnosis and treatment of tumors. Therefore, they have attracted attention in scientific research and clinical treatment (Cui and Xu, 2017; Goor et al., 2017; Guo et al., 2020; Cheng et al., 2021; Wang et al., 2022a). In modern cancer therapy, almost all drugs interact with their receptors *via* supramolecular characteristics. (Feng et al., 2017; Wang et al., 2023). Supramolecular chemistry gets inspiration from the living system and combines with modern medicine to “feed back” into the living system, giving rise to supramolecular cancer therapeutic biomaterials (Liu et al., 2017; Rajora et al., 2017; Rui et al., 2017; Sato et al., 2018; Chang et al., 2019; Yan et al., 2019; Ding et al., 2021; Tian et al., 2021; Lu et al., 2022a; Ding et al., 2022). Supramolecular cancer therapeutic biomaterials are an interdisciplinary field that focuses on the use of supramolecular chemistry to improve medicine by means of molecular recognition and assembly. (Brown and Anseth, 2017; Webber and Langer, 2017; Zhang et al., 2017; Zhang et al., 2021; Lu et al., 2022b). In particular, the advent of supramolecular cancer therapeutic biomaterials has provided new means to improve the pharmacokinetics of existing medications to provide more effective therapies, which is very beneficial for the treatment of cancer and other related diseases (Acar et al., 2017; Song et al., 2017; Zhu et al., 2018; Wang et al., 2022b; Guan et al., 2022). This paper focuses on the research of supramolecular biomaterials and discusses their design, synthesis, and characterization, in addition to their value in cancer treatment. This paper reviews the research progress of novel supramolecular hosts based on biomaterials and their application and efficiency in the field of cancer treatment.

Host-guest recognition is one well-known type of supramolecular interaction. Based on this, two supramolecular biomaterials (CB [7]⊃DOX and CB [7]⊃CPT) were constructed by Chen et al. After the creation of supramolecular biomaterials, the stability of DOX and CPT was significantly enhanced, while the chemotherapeutic drugs’ anticancer activities were maintained.

In addition to the cucurbit [7]uril-based host-guest interaction, Zhang et al. developed a class of high-performance nanodrugs based on supramolecular strategy and tested their performance. Through the CB [8] complex to deliver targeted drugs, they developed a class of PSMA-targeted supramolecular nanoparticles, in which doxorubicin (DOX) was encapsulated. The experimental study found that the level of DOX uptake by cells and the therapeutic effect had greatly improved.

Another type of supramolecular interaction is π-π stacking. By combining π-π stacking and pillar [5]arene-based interaction, Zhong et al. constructed luminescent biomaterials from a thiophene-based α-cyanostyrene-derivative (TPPA). It was found that TPPA can form nanoparticles in DMSO based on its self-assembly mode and will emit strong fluorescence during this process. However, after increasing the water ratio, the fluorescence intensity and the red shift somewhat decreased, and the self-assembly morphology also changed significantly and turned into fiber. When P5 and TPPA are combined to form a host-guest complex during the experiment, a white light will be released, which can be applied in cancer cell imaging.

Designing and synthesizing new macrocyclic molecules is very important for enhancing supramolecular cancer therapeutic biomaterials. In view of this, Wu et al. developed a TPE-based tetracationic cyclopropane. The experimental results show that this material can capture intracellular NADPH, improve the antioxidant capacity of cancer cells, and reduce ROS toxicity in cancer cells. When the concentration of GSH in cells decreases, this substance can act as a GSH response fluorescence switch, thus improving image quality and displaying cells clearly.

Modification of nanomaterials through supramolecular interactions is an important way to prepare supramolecular biomaterials. Lv et al. have highlighted the strategies for treating myocardial infarction (MI) using supramolecular biomaterials. These strategies include cardiac targeting drug delivery, an antiapoptosis strategy, and supramolecular biomaterials-mediated stem cell therapy. In addition, supramolecular biomaterials-based diagnosis strategies for MI were presented in terms of supramolecular biomaterials-based immunoassays. In short, this material indicates broad application prospects in the diagnosis and treatment of myocardial infarction, although there are still many problems to be solved. This should serve as the basis for the development of a more effective myocardial infarction treatment technology.

Mesoporous silicon nanomaterials can have their biodegradability greatly improved through supramolecular modification. In view of this, Duan et al. prepared a lymphatic targeting imaging agent called ICG@HMONs-HA, based on an indocyanine green-mesoporous silicon system. ICG@HMONs-HA can target lymph vessels and exhibits a long residence time, which is extremely beneficial for this kind of fluorescence imaging and the clinical translation of nanomaterial-based tracers.

Supramolecular polymers are excellent precursors for producing supramolecular biomaterials. Waterborne polyurethane is widely used in the preparation of supramolecular hydrogels, which can be used as an ideal additive to improve the mechanical properties of supramolecular hydrogels. Shen et al. introduced a large amount of waterborne polyurethane during experimental research and prepared an acrylamide supramolecular hydrogel under specific reaction conditions. The detection results showed that the viscosity of this gel reached a very high level. This is mainly because the polyurethane emulsion can form strong chemical crosslinks and produce strong interactions in polyurethane microregions. Using this method lowers the cost of crosslinked hydrogel, and the operation is convenient, so it has wide application prospects in the preparation of supramolecular biomaterials.

In conclusion, we greatly appreciate the efforts, insights, and visions of all contributors to the field of supramolecular cancer therapeutic biomaterials and hope that this Research Topic will provide perspective on using supramolecular chemistry to solve specific biomedical problems and will encourage further research in this field.
